# Editorial: Lead Risk Assessment and Health Effects

**DOI:** 10.3390/ijerph13060587

**Published:** 2016-06-14

**Authors:** Howard W. Mielke

**Affiliations:** Environmental Signaling Group, Department of Pharmacology, School of Medicine, Tulane University, New Orleans, LA 70112, USA; hmielke@tulane.edu; Tel.: +1-504-988-3889

**Keywords:** global lead issues, air lead, soil lead, water lead

## Abstract

In 1980, Clair C. Patterson stated: “Sometime in the near future it probably will be shown that the older urban areas of the United States have been rendered more or less uninhabitable by the millions of tons of poisonous industrial lead residues that have accumulated in cities during the past century”. We live in the near future about which this quote expressed concern. This special volume of 19 papers explores the status of scientific evidence regarding Dr. Patterson’s statement on the habitability of the environments of communities. Authors from 10 countries describe a variety of lead issues in the context of large and small communities, smelter sites, lead industries, lead-based painted houses, and vehicle fuel treated with lead additives dispersed by traffic. These articles represent the microcosm of the larger health issues associated with lead. The challenges of lead risk require a concerted global action for primary prevention.

Geochemist Clair C. Patterson became distressed about the health impacts of the massiveness of the 20th century quantities of lead in commerce and their residues [1]. This topic remains important for comprehending the present status of human health and welfare. Multiple advances in science serve to refine our understanding about the extent of lead in the environment and the health effects it poses. The articles in this volume provide a window into what is currently known about the topic and join tens of thousands of articles on the topic. They underscore recent advances and the global extent concerning the environmental and public health outcomes of lead exposure.

The theme of lead risk assessment and health effects was explored and interpreted by the authors of 19 manuscripts. The papers reflect the international scope of lead research conducted in Australia [2,3], Benin Sub-Saharan West Africa [4], Brazil [5], China including Taiwan [6,7,8,9,10,11,12], France [13], Mongolia [14] Singapore [15], South Africa [16,17], The Netherlands [18], and the USA [19,20]. The authors submitted articles on a wide range of lead risk assessment topics. One article is on the risk assessment of fluorides [11] included because of the negative impact that fluorides have on calcium metabolism which plays a role in the biochemistry of lead. The articles go beyond lead-based paints and include topics about the entire set of environmental components, water [15,19], air (dust) [2,3,6,12,14], and soil [6,18,19,20]. There are articles covering spatial and temporal relationships between lead-containing products and delayed health impacts such as motor neuron diseases [3]. The scope of lead effects includes: the association between women and infant exposure including breast milk [4,16]; lead worker exposure [5,7]; seasonality of exposure risk [4,19]; meta-analysis as a tool for data treatment [8]; and the mapping of community soil lead [18,20]. Acknowledging the gamut of exposure pathways and the effects of lead on society should advance the understanding about the need to promote the use of use skills and tools for primary prevention.

The critical issue at this point is the need to revitalize primary prevention and develop skills and tools to curtail the risks of lead exposure. The most vulnerable individuals to the risks of lead exposure are children and because the exposure appears without symptoms this makes the issue invisible. One tendency is to rely on children’s blood lead levels as a tool for discovery of environmental lead. This is not primary prevention. Unexpected results continue to mount regarding the long-term health outcomes associated with ever smaller amounts of lead exposure during early stages of development [21].

Along with policy matters there are scientific barriers that thwart primary prevention progress. Measurement techniques are critical for addressing environmental lead and human exposure, and instrumentation developments, such as hand-held field X-ray Fluorescence, have allowed major strides toward identifying sources of lead in the environment. However, in the case of soil there is a critical issue apropos the difference between lead content and *lead loading*. Within interior environments the practice of measuring the lead content of the vacuum cleaner bag dust was discarded in favor of floor wiping methods that measure *lead loading*. The methodology change occurred because there was a stronger association between *lead loading* and blood lead than between lead content and blood lead. In the outside soil environment, lead content is the standard method of measurement. However, an improved understanding about the quantity of lead in the environment could be achieved by recognizing the amount of *lead loading* of the soil surface; *lead loading* of soil generally exceeds by large factors the current standard for interior floor surfaces (which some consider too high) [22].

Another scientific issue involves the concept of *margin-of-safety*. The engineering and toxicology/pharmacology professions recognize the need to apply *margin-of-safety* factors to protect human health within the context of their respective trades. The *margin-of-safety* concept has not been included in the standards for lead and the outcome has been a continuing series of failures to protect children [21]. Given the current understanding that there is no known safe level of lead exposure for children, then a *margin-of-safety* factor is an essential addition to the standards to make them relevant for protecting children [22]. Primary prevention must proceed to protect the children of the global society and one hope for this small volume is that it contributes toward that outcome.
